# Evaluating therapeutic efficacy of iopanoic acid in a DMM-induced osteoarthritis mouse model and osteochondral lesioned human explants

**DOI:** 10.1016/j.ocarto.2026.100833

**Published:** 2026-06-05

**Authors:** S. Sana Sayedipour, Giorgia Mazzini, Margo Tuerlings, Jelle Nikkels, Marijke Koedam, Luis J. Cruz, Rachid Mahdad, Louise de Weerd, Bram van der Eerden, Yolande FM. Ramos, Ingrid Meulenbelt

**Affiliations:** a Department of Radiology, Leiden University Medical Center, 2333 ZA Leiden, the Netherlands; b Department Biomedical Data Sciences, Molecular Epidemiology, Leiden University Medical Center, 2333 ZA Leiden, the Netherlands; c Department of Internal Medicine, Erasmus MC, Erasmus University Medical Center, 3015 GD, Rotterdam, the Netherlands; d Department of Orthopaedics, Alrijne Hospital, Leiderdorp, the Netherlands; e Departments of Radiology & Human Genetics, Leiden University Medical Center, 2333 ZA Leiden, the Netherlands

**Keywords:** Osteoarthritis, DMM *in vivo* OA model, Thyroid hormones, IOP, Lesioned osteochondral human explants

## Abstract

**Objective:**

To evaluate the therapeutic potential of iopanoic acid (IOP), a thyroid hormone pathway inhibitor, in preserving cartilage and bone integrity in osteoarthritis (OA), using *in vivo* and *ex vivo* tissue models.

**Design:**

In the DMM mouse model, IOP was administered through intra-articular (i.a.) injection, either alone or combined with a thermosensitive hydrogel to enable sustained release. Histological analyses included damage, osteophyte, and synovitis scoring. Immunohistochemistry was performed for Col2, Mmp13, and CCDC80 to evaluate anabolic, catabolic, and hypertrophic markers. Micro-CT assessed subchondral bone changes. In the *ex vivo* studies, IOP was applied to lesioned human osteochondral OA explants. Matrix degradation and repair were evaluated by sulfated glycosaminoglycan (sGAG) release, Mankin histology scores, and RT-qPCR for cartilage matrix genes.

**Results:**

Administration of IOP significantly reduced cartilage degeneration in DMM mice, characterized by increased Col2, and decreased Mmp13 and CCDC80 expression. Notably, IOP also prevented pathological subchondral bone thickening. In human explants, IOP treatment led to a significant reduction in sGAG release compared to untreated explants on day 6 of the IOP treatment. Moreover, Mankin scores were significantly improved in IOP-treated compared to untreated explants, indicating reduced cartilage degradation.

**Conclusion:**

IOP demonstrates strong chondroprotective effects, reducing cartilage degradation and promoting repair in OA models. Its combination with a thermosensitive hydrogel amplifies therapeutic potential, offering a promising strategy for OA treatment. Next steps are to optimize delivery and validate early molecular effects.

## 1 Introduction

Osteoarthritis (OA) is a prevalent, complex, disabling disease of articular joints, characterized by degradation of articular cartilage and remodeling of the subchondral bone [1]. Despite extensive research, no treatment has been found that can alter the course of the disease [2]. In order to develop innovative effective treatments, a deeper understanding of the underlying mechanisms of OA disease processes is essential [3,4]. To gain such insights, genetic studies have been performed and have highlighted a significant role for genes involved in regulating chondrocyte transitions during endochondral ossification [[4], [5], [6]]. One such gene is *DIO2*, encoding enzyme deiodinase iodothyronine type 2 (D2) that converts intracellular inactive thyroxine (T4) into its active form, triiodothyronine (T3), thereby increasing intracellular T3 bioavailability. Notably, T3 signaling is known to initiate terminal chondrocyte maturation in growth plate tissues hence inducing hypertrophy and leading to cartilage breakdown and mineralization [7,8]. By applying functional follow-up studies of the *DIO2* gene in articular cartilage, it was shown that upregulation of *DIO2* expression, particularly modified by mechanical loading [8,9] affected articular cartilage integrity. Additionally, other studies have shown that a minor allele of the *DIO2* gene (Ala92) impairs the conversion of the prohormone T4 to the active hormone T3 and is controversially associated with osteoarthritis [10]. Subsequently, *in vitro* [8] and *in vivo* [11] models were applied to demonstrate that attenuation of D2-enzyme activity by Iopanoic acid (IOP) could effectively prevent chondrocyte terminal maturation. Even more, we showed that IOP could have a beneficial effect to injurious mechanical stress inflicting OA-like damage in *ex vivo* aged human osteochondral explant [12]. To accommodate sensitive readout in such preclinical models we showed that *CCDC80* encoding Coiled-Coil Domain Containing 80 is a robust and sensitive marker of T3 induced chondrocyte terminal maturation [13]. Together, we have previously shown that targeted inhibition of D2, could help preserve cartilage homeostasis by restraining chondrocyte hypertrophy and maintaining matrix integrity [8].

To facilitate localized and sustained delivery of IOP for therapeutic use, we previously developed and characterized a thermosensitive hydrogel based on 25% poloxamer 407 (P407), which enables minimally invasive intra-articular injection and drug retention at the joint site [14]. This hydrogel has proven to be effective for encapsulating small molecules like IOP, enhancing local bioavailability while minimizing systemic exposure. Building upon this, here we set out to evaluate the therapeutic potential of IOP in reducing OA-related damage using two complementary models: the *in vivo* destabilization of the medial meniscus (DMM) mouse model and *ex vivo* lesioned OA human osteochondral explants. The DMM model effectively replicates mechanical instability-induced OA progression in humans, providing a reliable platform for studying cartilage degradation and testing interventions [15]. In parallel, human OA explants retain pathophysiological features of diseased cartilage, enabling direct evaluation of therapeutic responses in clinically relevant tissues. This combined approach provides a robust framework to test IOP's potential in modulating chondrocyte activity, preserving the cartilage extracellular matrix (ECM), and maintaining cartilage integrity in OA.

## 2 Methods

### 2.1 *In vivo* study design

The animal procedures were all conducted at the Leiden University Medical Center and were approved by the Animal Welfare Committee (IvD) under number AVD1160020171405 - PE.18.101.006 and in line with ARRIVE guidelines 2.0.28 mice in total has been conducted within the experiment. All mice were housed in groups in polypropylene cages on a 12-h light/dark cycle with unrestricted access to standard mouse food and water. Surgical DMM was performed on the right knee joint in male C57BL/6J mice at 12 weeks old, as described before [15,16]. Detail of animal experiment and procedures are described in the **Supplementary Materials and methods**. After surgery, mice had access to food and tap water. 21 days after DMM surgery, mice were randomly and equally divided into five groups (Sham: n = 4; DMM: n = 6 in each group). They received one-time treatment via intra-articular (i.a.) injection. 35 days after i.a. administration of the treatment groups, mice were euthanized by continuous CO_2_ inhalation, the right knee joints were harvested and fixed with 4 % paraformaldehyde. To assess outcome parameters we used high-resolution micro computed tomography on trabecular bone, histology and immunohistology. To perform histological analysis, decalcification was performed using Mol-decalcifier (Milestone). Detailed description of Micro-CT, histological and immunohistochemical scoring is provided in the **Supplementary Materials and Methods**.

### 2.2 *Ex vivo* human lesioned explant experiment

#### 2.2.1 Study design and culture components

Osteochondral explants were collected from macroscopically preserved and lesioned areas from joints obtained from OA patients in the RAAK study [17]. The RAAK-study is aimed at biobanking of joint materials of patients who underwent a total joint replacement surgery due to OA. The RAAK-study is approved by the medical ethics committee of the Leiden University Medical Center (P19.013) and informed consent was obtained from subjects.

A total of 38 osteochondral explants were obtained from six donors for this study and were divided into treatment groups as follows: control preserved (n = 10 explants, N = 6 donors), lesioned (n = 11 explants, N = 6 donors), and lesioned with IOP (n = 16 explants, N = 6 donors). Donor characteristics are provided in Supplementary Table S2. After harvesting, the explants were first washed with PBS and taken into culture in chondrogenic differentiation medium in a 5 % CO_2_ incubator at 37 °C. The medium was refreshed every three days measurements.

#### 2.2.2 IOP treatment

IOP treatment for the lesioned explants began on day 3, with 100 μM of IOP added [12] to the medium on days 3, 6, and 9. On day 12, cartilage and bone were separated using a scalpel, snap frozen in liquid nitrogen and stored at 80 °C for RNA isolation. For histology, a part of cartilage was fixed in 4 % formaldehyde. Medium was collected on day 12 and stored at 80 °C. Details on Sulfated glycosaminoglycan (sGAGs) measurement, histology and RNA isolation, Reverse Transcription and quantitative Real-Time PCR can be found in **Supplementary Materials and methods**.

#### 2.2.3 Statistical analysis

Statistical analyses were performed using IBM SPSS Statistics 25. Data are presented as mean ± standard deviation of 4–6 independent experiments, unless otherwise stated. For the *in vivo* experiments, generalized estimating equations (GEE [12]) were applied to independently evaluate the effects of the hydrogel and IOP treatment. For the *ex vivo* experiments, including histology, sulfated glycosaminoglycan (sGAG) release, and gene expression, differences between lesioned control and treated explants were analyzed using linear GEE with robust variance estimators to account for dependencies between donors. Donor was included as a clustering variable to correct for within-donor correlations. The Beta coefficients derived from the GEE models represent the estimated differences between treated and lesioned control explants. Corresponding *P*-values were obtained from the same models. A *P*-value ≤0.05 was considered statistically significant and is indicated as follows: ∗*P* ≤ 0.05, ∗∗*P* ≤ 0.01, ∗∗∗*P* ≤ 0.001, ∗∗∗∗*P* ≤ 0.0001.

## 3 Results

### 3.1 Therapeutic potential IOP in *in vivo* DMM mouse model on joint tissue damage

**Fig. 1:**
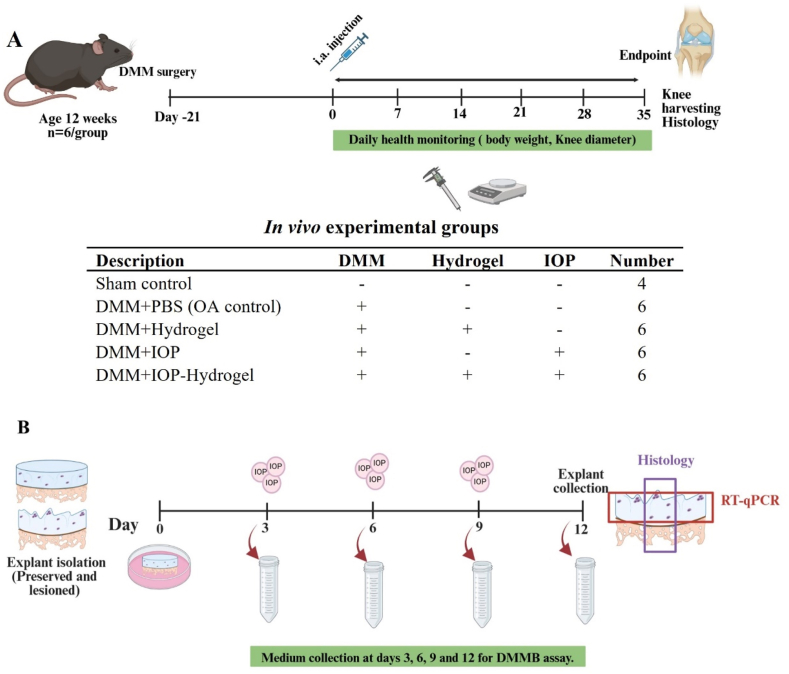
Experimental timeline: (A) *In vivo* OA model. 21 days before start treatment, we induce OA by DMM. At day 0, treatment groups (PBS, Hydrogel, IOP ± hydrogel) injected i.a. in to the mice knee. 35 days after injection, mice were sacrificed and their right knee collected to perform histology. (B) *Ex vivo* experiment: Macroscopically preserved and lesioned osteochondral explants were punched from the human OA knee and taken into culture. Lesioned osteochondral explants were treated with IOP (100 μM) from day 3 onwards. Media was collected at the indicated days and subsequently each explant received fresh media. Finally, on day 12, explants harvested, one section was fixed in 4 % formaldehyde for histology, while for the remaining explant cartilage and bone were separated, snap frozen and stored at −80 °C for downstream analyses. DMM = Destabilization of the Medial Meniscus; i.a. = Intra articular; OA= Osteoarthritis; IOP= Iopanoic acid.

In Fig. 1 and Supplementary Table S1 a schematic overview is presented of our experimental strategy to determine the therapeutic efficacy of IOP treatment. Three weeks after DMM surgery mice received a single intra articular (i.a.) injection with PBS, IOP, hydrogel or IOP + hydrogel randomly assigned to the mice (Fig. 1A). Six weeks after i.a. injections cartilage integrity was evaluated Safranin-O fast green staining.

**Fig. 2:**
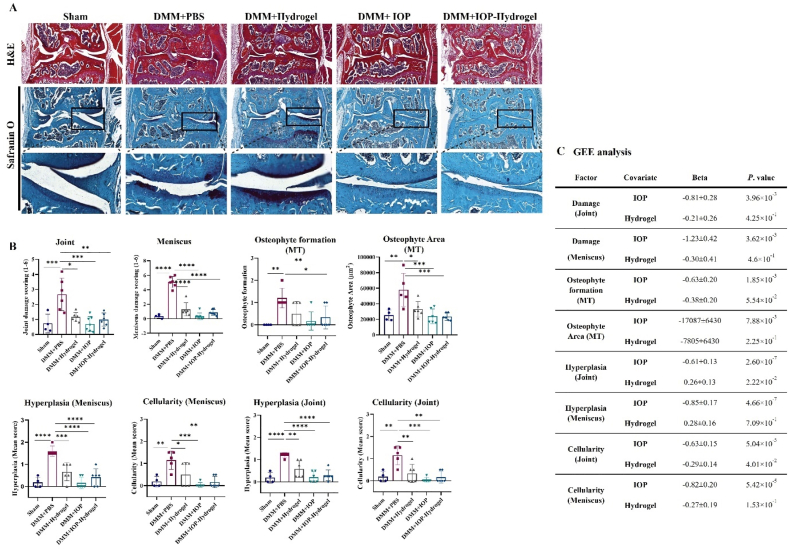
Evaluation of the therapeutic effects of the IOP and hydrogel treatment in the DMM induced OA mouse model. Experimental treatments were administered by a single intra-articular (i.a.) injection 3 weeks after DMM surgery.(**A**) Knee joints were harvested 9 weeks after DMM surgery and analyzed histologically using H&E and Safranin O/Fast Green staining. Scale bars, 40 μm. (B) Quantification of joint damage, meniscus damage, osteophyte formation in the medial tibia (MT), osteophyte area, and synovitis-related parameters including synovial hyperplasia and sublining cellularity in both the joint and meniscal regions. Joint and meniscus lesions were graded using the structural cartilage damage scoring system on a scale of 0–6. Osteophyte formation was scored semi-quantitatively based on osteophyte presence at the inner and/or outer joint margins. Synovitis was evaluated based on two parameters: synovial lining hyperplasia and sublining cellularity, scored semi-quantitatively on a scale of 0–3, while osteophyte area was quantified in μm^2^ using ImageJ. (C) GEE analyses were performed to determine the independent therapeutic effects of IOP and hydrogel. All data presented as mean ± standard deviation (n = 4–6). Statistically significant differences are indicated by ∗*P* ≤ 0.05, ∗∗*P* ≤ 0.01, ∗∗∗*P* ≤ 0.001, and ∗∗∗∗*P* ≤ 0.0001 between the indicated groups. DMM = Destabilization of the Medial Meniscus; H&E = Hematoxylin and Eosin; i.a. = Intra-articular; MT = Medial Tibia; GEE = Generalized Estimating Equation.

As shown in Fig. 2A, safranin-O and H&E staining in positive control mice (DMM + PBS) showed markedly disrupted cartilage architecture, with patchy and irregular staining reflecting substantial proteoglycan loss and matrix degradation, accompanied by roughened cartilage surfaces and evident cracks. The disrupted cartilage architecture in the positive control mice was accompanied by an average joint and meniscus damage score of 2.66 ± 1.07 and 5.06 ± 0.77, respectively (Fig. 2B). Notably, treatment with IOP and/or hydrogel resulted in improved cartilage integrity, with smoother articular surfaces and more uniform matrix staining, with statistically significant lower average joint and meniscus damage scores between 0.023 - 0.26 and 0.51–0.94 reaching negative control (no DMM, sham) levels. To further dissect the independent contributions of IOP and hydrogel on joint and meniscus damage, a multi-variate regression analysis was performed (Fig. 2C). The analysis demonstrated a significant protective effect of IOP on joint damage (Beta = −1.24, *P* = 3.11 × 10^−5^) and meniscus damage (Beta = −1.86, *P* = 2.42 × 10^−5^). Hydrogel treatment also showed a significant, though smaller, protective effect on both joint damage (Beta = −0.61, *P* = 3.40 × 10^−2^) and meniscus damage (Beta = −0.88, *P* = 3.65 × 10^−2^). These findings indicate that both IOP and hydrogel contribute to reducing OA-associated structural damage, with IOP exerting the stronger protective effect.

Next, we scored osteophytes in the medial tibia compartment (MT) and synovial inflammation in the meniscal region (Meniscus) and joint capsule (Joint), based on synovial lining hyperplasia and sublining cellularity. As shown in Fig. 2B positive control mice (DMM + PBS) had modest osteophytes at both inner and outer joint margins, with a score and osteophyte area reaching 1.20 ± 0.84 and 57272 ± 29182 μm^2^, respectively. Moreover, positive control mice exhibited some synovial hyperplasia and inflammatory cellular infiltration in both the Joint and meniscal regions with scores 1.13 ± 0.11 and 1.58 ± 0.42, respectively. The cellularity scores in meniscus and Joint reached 1.00 ± 0.53 and 1.13 ± 0.34. As shown in Fig. 2B, treatment with IOP and/or hydrogel showed an overall reduction in osteophyte severity, osteophyte area, synovial hyperplasia, and cellular infiltration, with values approaching sham control levels. Multivariate regression analysis (Fig. 2C) further confirmed a statistical significant independent reduction in osteophytes and synovial inflammation of IOP. Although the hydrogel also showed a modest reduction in osteophytes and synovial inflammation, the effects did not reach statistical significance.

### 3.2 Therapeutic effect of IOP in *in vivo* DMM mouse model by immunohistochemistry for Col2, Mmp13, and CCDC80

**Fig. 3:**
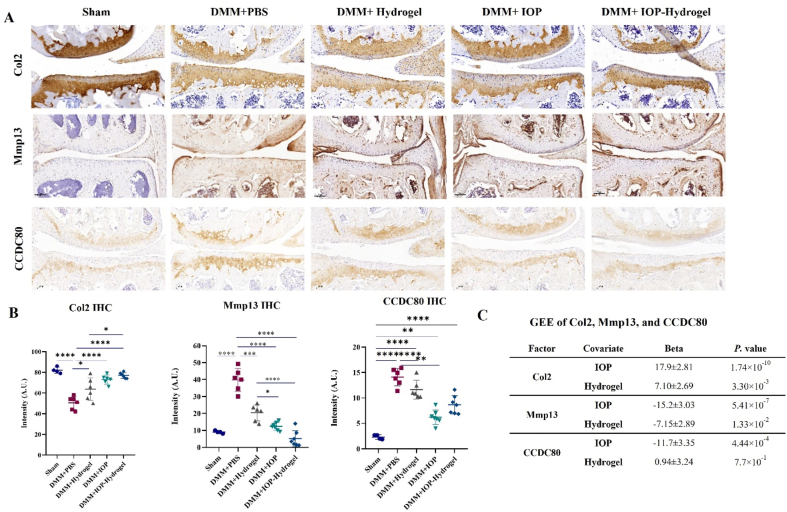
Immunohistochemistry staining of Col2, Mmp13, and CCDC80 of the medial condyle cartilage at 9 weeks after the DMM procedure. (A) Representative IHC images of Col2, Mmp13, and CCDC80 staining in meniscus lesions of the knee joint. (B) Quantification of IHC staining intensity for Col2, Mmp13, and CCDC80 using ImageJ-based image analysis. (C) Multivariate analyses of Col2*,* Mmp13 and CCDC80 using GEE to understand the independent therapeutic effect of IOP and hydrogel in the meniscus lesions. All data are shown as means ± standard deviations (n = 6). Statistically-significant differences are indicated by ∗*P* ≤ 0.05, ∗∗*P* ≤ 0.01, ∗∗∗*P* ≤ 0.001, ∗∗∗∗*P* ≤ 0.0001. GEE: Generalized Estimating Equation.

To further investigate the therapeutic effects of IOP and/or hydrogel treatment on cartilage homeostasis, we performed immunohistochemical (IHC) staining for Col2 an anabolic ECM protein, Mmp13 a catabolic protease involved in matrix breakdown, and CCDC80 a marker of terminal chondrocyte differentiation. Representative staining images are shown in Fig. 3A and associated quantified staining intensity in (Fig. 3B). In the positive control mice (DMM + PBS), Col2 staining was markedly reduced, indicating substantial matrix loss, whereas Mmp13 and CCDC80 signals were strongly elevated throughout the deep cartilage and subchondral regions, reflecting enhanced catabolic and hypertrophic activity. Among treatment groups (IOP, hydrogel, or IOP + hydrogel) we showed partially restored Col2 deposition, and reduced both Mmp13 and CCDC80 expression. Herein, the combined IOP + hydrogel group displayed most robust chondroprotective effects with dense and continuous Col2 distribution, and markedly reduced Mmp13 and CCDC80 signals, closely resembling the sham phenotype.

Subsequent multi-variate regression analyses (Fig. 3C) confirmed that IOP significantly increased Col2 staining intensity (Beta = 17.9, *P* = 1.74 × 10^−10^), indicating a strong enhancement of cartilage matrix synthesis. Hydrogel also elevated Col2 (Beta = 7.10, *P* = 3.30 × 10^−3^), though the effect was less pronounced than with IOP. Both treatments reduced Mmp13 expression, with IOP having a stronger effect (Beta = −15.2, *P =* 5.41 × 10^−7^) than hydrogel (Beta = −7.15, *P* = 1.33 × 10^−2^), indicating that both interventions inhibit catabolic activity in OA cartilage. Regarding CCDC80, IOP significantly suppressed expression (Beta = −11.7, *P* = 4.44 × 10^−4^), suggesting reduced terminal maturation, while hydrogel showed no significant effect. These results highlight IOP's role in promoting anabolic repair and limiting catabolic and hypertrophic processes in OA cartilage.

### 3.3 Therapeutic effect of IOP treatment in *in vivo* DMM mouse model on subchondral bone remodeling

**Fig. 4:**
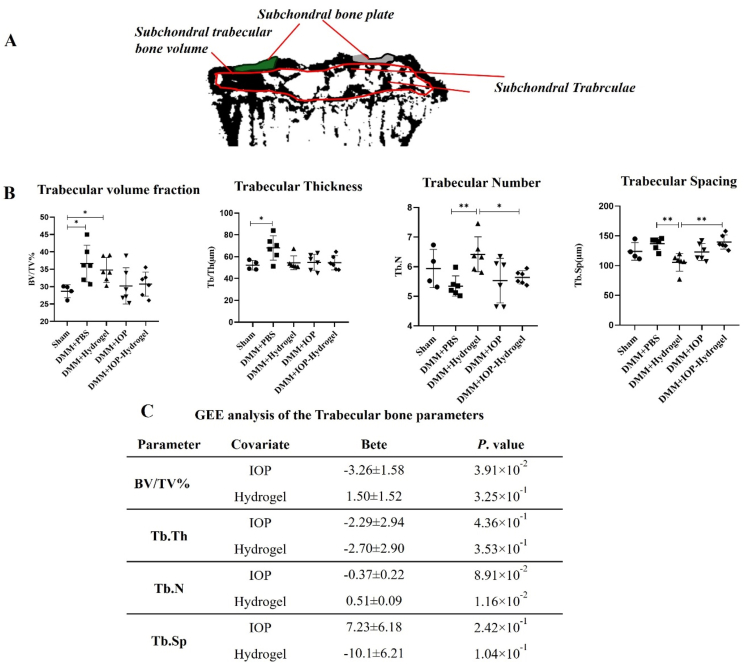
Micro computed tomography analysis of subchondral bone changes following IOP and hydrogel treatment in the DMM model. (A) Representative micro-CT images showing the defined region of interest in the medial tibial plateau for trabecular bone analysis (highlighted in red). (B) Quantitative assessment of trabecular bone parameters, including bone volume fraction (BV/TV), trabecular thickness (Tb.Th), trabecular number (Tb.N), and trabecular spacing (Tb.Sp) across treatment groups. (C) GEE analysis indicating the effects of IOP and hydrogel on trabecular bone parameters. Statistically-significant differences are indicated by ∗*P* ≤ 0.05, and ∗∗*P* ≤ 0.01 between the indicated groups. GEE: Generalized Estimating Equation.

Micro computed tomography analysis was conducted to quantitatively evaluate subchondral bone remodeling in the tibial plateau of mice. The region of interest was defined in the subchondral trabecular bone, as illustrated in representative micro-CT images (Fig. 4A), and analyzed using standard trabecular parameters. Fig. 4B presents quantitative micro-CT data of trabecular parameters. Compared to the sham group, the DMM + PBS group exhibited notable deterioration in trabecular bone structure. Specifically, there was a significant increase in bone volume fraction (BV/TV%) (*P* = 2.28 × 10^−2^) and trabecular thickness (Tb.Th) (*P* = 2.81 × 10^−2^). Although trabecular number (Tb.N) showed a decrease and trabecular separation (Tb.Sp) also increased in the DMM + PBS group, these changes did not reach statistical significance. Treatment with IOP, either alone or in combination with hydrogel, normalized all measured trabecular parameters, with no statistically significant differences compared to the sham group. Notably, hydrogel-based treatments alone demonstrated additional effects. The DMM + hydrogel group showed a significant increase in Tb.N (*P* = 8.50 × 10^−3^) and a significant decrease in Tb.Sp (*P* = 3.20 × 10^−3^) compared to the DMM + PBS group, indicating partial restoration of the trabecular microarchitecture. Moreover, the DMM + IOP - hydrogel group exhibited a significant decrease in Tb.N (*P* = 1.30 × 10^−2^) and a significant increase in Tb.Sp (*P* = 1.30 × 10^−3^) when compared to the DMM + hydrogel group, suggesting that IOP contributed to a normalizing effect, bringing these parameters closer to those observed in the sham group. Further evaluate the independent effects of hydrogel and IOP treatments, a GEE analysis was conducted (Fig. 4C). Based on this analysis, both treatments appeared to normalize changes in subchondral bone parameters upon DMM-induced damage (Fig. 4C). IOP treatment significantly reduced BV/TV% (Beta = −3.26; *P* = 3.91 × 10^−2^), indicating strong suppression of OA-associated subchondral bone thickening. In contrast, hydrogel treatment significantly increased trabecular number (Tb.N) (Beta = 0.51; *P* = 1.16 × 10^−2^).

### 3.4 Therapeutic effect of IOP treatment in human lesioned OA osteochondral explants

**Fig. 5:**
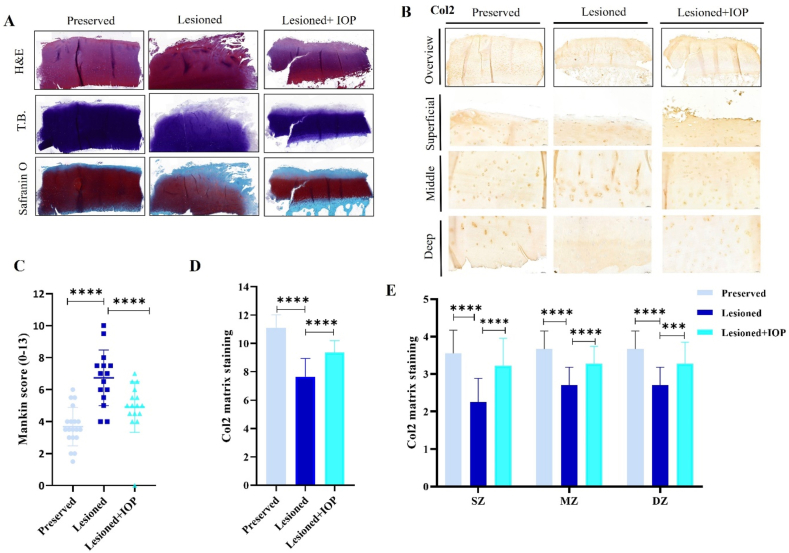
Histological evaluation and collagen type II (Col2) immunohistochemistry of preserved, lesioned, and IOP-treated lesioned human osteochondral explants. (A) Representative histological images of H&E, Toluidine Blue, and Safranin O -fast green staining. (B) Representative Col2 immunohistochemical staining showing overview sections and higher magnification images of the superficial, middle, and deep cartilage zones. (C) Quantification of cartilage degradation using the Mankin scoring system. (D) Quantification of CoL2 matrix staining across the cartilage and (E) Quantification of CoL2 matrix staining in superficial, middle, and deep cartilage zones. Col2 expression was scored by two independent blinded observers based on cytoplasmic and nuclear staining of chondrocytes in the superficial, middle, and deep cartilage layers. Staining intensity was graded as 0 (no staining), 1 (moderate staining), or 2 (strong staining). Regression analysis indicating the effects of IOP treatment. Statistically-significant differences are indicated by ∗*P* ≤ 0.05, ∗∗*P* ≤ 0.01, ∗∗∗*P* ≤ 0.001, and ∗∗∗∗*P* ≤ 0.0001 between the indicated groups. H&E = hematoxylin and eosin; T.B = Toluidine Blue; Col2 = Collagen type II; SZ: superficial zone; MZ: middle zone; DZ: deep zone.

To address the translational potential of IOP treatment in human age-related osteoarthritis, we next aimed to study its chondroprotective effect in lesioned osteochondral explants, from OA patients undergoing a joint replacement surgery, following the schedule outlined in Fig. 1B. In doing so we compared human lesioned OA articular cartilage with and without IOP treatment while preserved articular cartilage was used as an additional reference. Twelve days after IOP treatments started, histological evaluation was performed using Mankin scoring together with Col2 immunohistochemistry. As shown in Fig. 5A and B, preserved explants displayed well-organized cartilage architecture with uniform and intense staining across H&E, Toluidine Blue, and Safranin O–Fast Green, representing intact ECM with an average Mankin-score of 3.68 ± 1.20. In contrast, non-treated lesioned explants showed substantial cartilage damage, characterized by disrupted tissue architecture, reduced proteoglycan staining, and surface fibrillation, confirming severe matrix degradation, with an average Mankin-score of 6.73 ± 1.74. Notably lesioned explants treated with IOP + gel demonstrated significant restoration of cartilage integrity, with an average Mankin-score of 4.93 ± 1.63. Subsequent statistical analyses confirmed that IOP-treated lesioned explants revealed a statistically significant reduction in cartilage damage (Beta = −1.61 ± 0.6, *P* = 1.20 × 10^−2^), confirming the therapeutic effect of IOP in reducing structural degeneration in human OA cartilage. Assessment of cartilage matrix preservation by Col2 expression (Fig. 5C and D) showed that the markedly reduced Col2 staining in lesioned articular cartilage, was significantly restored across cartilage layers upon IOP treatment (Col2 SZ: Beta = 1.03 ± 0.12, *P* = 1.89 × 10^−8^; Col2 MZ: Beta = 0.87 ± 0.21, *P* = 4.79 × 10^−4^; Col2 DZ: Beta = 0.87 ± 0.23, *P* = 6.97 × 10^−3^), suggesting partial restoration of cartilage matrix integrity.

**Fig. 6:**
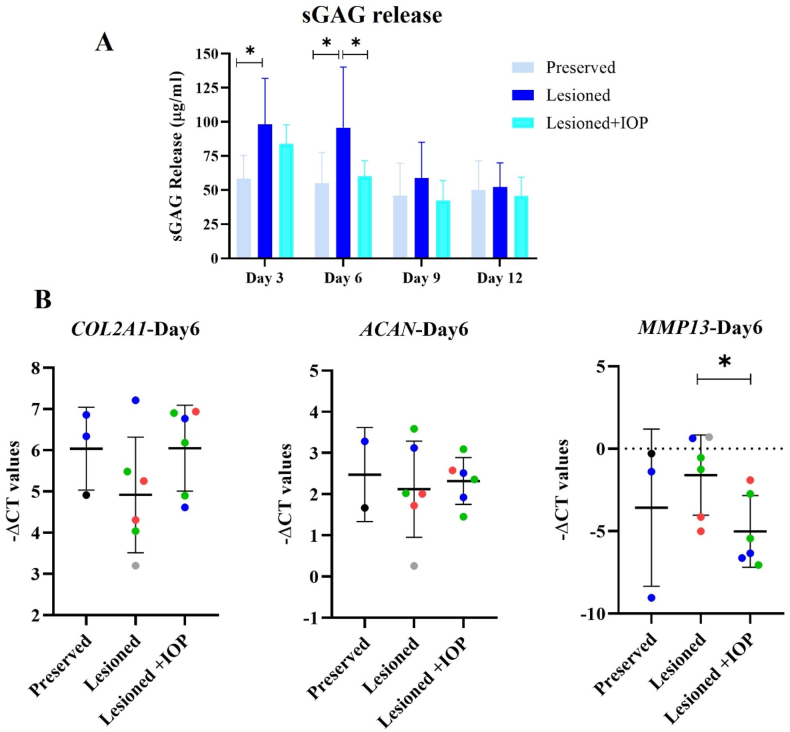
Assessment of sGAG release and gene expression in preserved, lesioned, and IOP-treated human osteochondral explants .(A) sGAG release measured by the DMMB assay at days 3, 6, 9, and 12 from preserved, lesioned, and IOP-treated lesioned explants. (B) Gene expression analysis by RT-qPCR at day 6 for *COL2A1*, *ACAN*, and *MMP13*, presented as −ΔCT values for preserved, lesioned, and IOP-treated lesioned explants. *P*-values of mean differences between groups were determined by regression analysis. Statistically significant differences are indicated by ∗ where *P* ≤ 0.05, ∗∗*P* ≤ 0.01 between the indicated groups.

Finally we studied the effect of IOP on ongoing matrix degradation during culturing using sGAG release. As shown in Fig. 6A, lesioned OA explants released markedly higher levels of sGAGs compared to preserved explants, with the highest levels observed on day 3, indicating ongoing matrix degradation. Notably, in the IOP-treated lesioned explants, we showed a reduction in sGAG release that with the largest difference at day-6. From day-6 onwards, sGAG levels gradually declined and stabilized in all groups. Statistical analysis confirmed a modest, statistically significant reduced sGAG release in IOP treated lesioned OA explants relative to untreated lesioned explants at day-6 (Beta = −38.91 ± 17.2, *P* = 4.03 × 10^−2^), indicating effective reduction of cartilage matrix degradation within 3–6 days post-treatment. By day 12, this difference was no longer significant, likely due to overall matrix stabilization in long-term culture with nutrient-rich medium.

To further evaluate the effects of IOP treatment on human chondrocyte phenotype, gene expression analyses of key anabolic (*COL2A1*, *ACAN*) and catabolic (*MMP13*) cartilage genes was performed. As shown in Fig. 6B we showed at day-6 a modest restoration of *COL2A1* and *ACAN* expression in IOP treated lesioned explants, albeit not statistically significant. Nonetheless, at day-6 we showed a statistically reduced expression of *MMP13* in IOP-treated lesioned articular cartilage compared to untreated lesioned controls (Beta = −2.71 ± 1.31, *P* = 3.90 × 10^−2^), in line with the sGAG release. No significant differences in the expression of these genes were detected among lesioned, and IOP-treated lesioned explants at other timepoints (Supplementary Fig. S1).

## 4 Discussion

This study investigated the therapeutic potential of inhibiting D2 activity using IOP, in both an *in vivo* DMM-induced OA mouse model and an *ex vivo* human osteochondral lesioned explant model. IOP treatment significantly reduced cartilage degradation in the DMM mouse model, as shown by lower damage score, decreased expression of catabolic marker Mmp13 and the hypertrophic marker CCDC80 while Col2 levels increased. In addition, IOP treatment reduced osteophyte formation and osteophyte area, while also attenuating synovial hyperplasia and inflammatory cellular infiltration, indicating broader protective effects on OA-associated joint remodeling and inflammation. Similarly, in the *ex vivo* human explant model, IOP treatment of lesioned OA explants improved cartilage integrity as reflected by histology, sGAG release and gene expression data. Together, these results confirm that pharmacological inhibition of *DIO2* effectively limits cartilage damage and supports matrix preservation in OA.

Mechanistically, these findings highlight the critical role of thyroid hormone activation in driving OA pathogenesis. Notably in this respect is *CCDC8*0 that was previously identified as a sensitive T3-responsive marker of terminally maturing chondrocytes [13]. The fact that IOP treatment reduced *Ccdc80* in the DMM mice is therefore further linking thyroid hormone signaling to ECM remodeling and cartilage deterioration [13,18,19]. Finally it is important to note that IOP was able to restore aspects of cartilage health in human lesioned OA explants from patients undergoing joint replacement surgery, indicating that IOP is able to directly induce a more quiescent and regenerative cellular state. Together, advocate that IOP treatment not only protects cartilage from structural deterioration as shown in the DMM mice model but also actively reshapes the chondrocyte phenotype toward a more regenerative, anti-hypertrophic state.

Beyond its chondroprotective role, IOP also modulated subchondral bone remodeling, another hallmark of OA progression. IOP treatment led to a significant reduction in bone volume, effectively counteracting the subchondral bone thickening typically associated with osteoarthritis progression. This aligns with the pathological features of OA, where early-stage disease is characterized by bone loss and cartilage degeneration, followed by excessive subchondral bone formation and osteophyte development in later stages [20]. Since thyroid hormones influence both osteoblast and osteoclast activity, *DIO2* inhibition may help normalize bone turnover and prevent aberrant bone formation [21]. While IOP alone led to variable trabecular parameters, co-delivery with hydrogel stabilized the bone microarchitecture and reduced inter-individual variability, supporting localized and sustained the therapeutic balance between cartilage preservation and bone remodeling. Although, the cargo release kinetics of IOP from the thermosensitive hydrogel were not directly assessed in this study, we based the timelines and concentration on our previous work using the same hydrogel system [14,22]. These studies showed that cargo release is primarily governed by hydrogel degradation. However, this remains to be confirmed, and future studies should include direct characterization of IOP release kinetics to better define its delivery behavior and therapeutic window.

In this study we applied a DMM model in male mice only, to sensitively mimic key aspects of OA pathophysiology, particularly post-traumatic mechanical instability-induced joint stress and progressive cartilage degradation [23]. As such by utilizing this model, we were able to observe the disease progression and evaluate the impact of D2 inhibition in a controlled *in vivo* environment for the first time. Nonetheless, as generally acknowledged the *in vivo* DMM mice model does not capture the metabolic and age-related components of idiopathic age-related human OA, occurring in male but with a preponderance in females. Nonetheless, the here applied human *ex vivo* osteochondral explant model, provided a complementary translational platform to assess IOP's effects. This because explants were collected from lesioned osteoarthritic cartilage of (male, female, and aged) patients undergoing a joint replacement surgery [24]. As such it reliably reflects human native cartilage–bone interface, chondrocyte organization, and ECM composition, allowing evaluation of IOP treatment in a clinically relevant human tissue context [25].

Despite the matrix-level improvements observed, gene expression analysis in human explant samples revealed only modes differences in the expression of key anabolic (*COL2A1*, *ACAN*) or catabolic (*MMP13*) markers across treatment groups. However, analysis of earlier time points demonstrated transient gene expression changes following cartilage injury and IOP treatment. At day 3, *ACAN* expression was significantly reduced in lesioned cartilage, reflecting an early suppression of anabolic matrix activity, while *COL2A1* expression remained largely unchanged. By day 6, IOP treatment significantly reduced the injury-induced increase in the catabolic marker *MMP13*, suggesting an early attenuation of catabolic responses.

While these findings support the therapeutic potential of IOP, a perceived weakness of our study is the fact that we did not include pain-related behavioral in mice nor included bone mineral density measurements in human explants. Moreover, it should be noted that the use of mol-decalcifier (Milestone), affected Safranin O staining to such an extent, that we could not reliably perform the more generally applied OARSI-scoring system. For that matter, cartilage degeneration was evaluated using a structural cartilage damage scoring system [26]. Since, we previously have shown that this damage scoring has strong correlation (r = 0.76) with OARSI grading [22], we are confident about the presented results in our manuscript. Finally, in the human *ex vivo* model, we could not exclude that the nutrient-rich culture conditions may have allowed partial spontaneous recovery, potentially underestimating treatment effects.

In summary, findings from both *in vivo* and *ex vivo* models demonstrate that IOP reduces cartilage degradation, supports tissue preservation, and modulates subchondral bone remodeling in OA. By limiting local thyroid hormone activation through D2 inhibition, IOP helps maintain cartilage homeostasis and reduces hypertrophic and catabolic signaling. These results highlight the translational potential of IOP as a disease-modifying OA therapy and emphasize the value of combining *in vivo* and *ex vivo* systems to bridge preclinical findings with human tissue–based evidence.

## Ethics approval and consent to participate

The project “Dose finding & potential adverse effects observations in the DMM model” was conducted at the Leiden University Medical Center and was approved by the Animal Welfare Committee (IvD) under number AVD1160020171405- PE.18.101.006 on Apr 21, 2023. Furthermore, the RAAK study has been approved by the medical ethical committee of the Leiden University Medical Center (P08.239/P19.013) and written informed consent was obtained from subjects.

## Consent for publication

Not applicable.

## Author contributions

Conceptualization: Sana S. Sayedipour, Yolande FM Ramos and Ingrid Meulenbelt. Methodology: Sana S. Sayedipour, Giorgia Mazzini, Margo Tuerlings, Jelle Nikkels, Marijke Koedam. Supervision: Ingrid Meulenbelt. Validation: Bram van der Eerden and Louise de Weerd. Writing – original draft: Sana S. Sayedipour and Ingrid Meulenbelt. Formal analysis: Sana S. Sayedipour, Ingrid Meulenbelt. Writing - Original Draft: Sana S. Sayedipour. Writing – reviewing and editing: Sana S. Sayedipour, Ingrid Meulenbelt, Giorgia Mazzini, Margo Tuerlings, Jelle Nikkels, Marijke Koedam, Luis J. Cruz, Richad Mahdad, Louise de Weerd, Bram van der Eerden, Yolande FM Ramos.

## Funding

This project has received funding of the Dutch Arthritis Society via the long term research programme (LLP32). and European Union's Horizon 2020 research and innovation program AutoCRAT under grant agreement No 874671. The material presented and views expressed here are the responsibility of the author(s) only. The EU Commission takes no responsibility for any use made of the information set out.

## Conflict of interests

Not declared.
