# Supplementary material for: Evaluating therapeutic efficacy of iopanoic acid in a DMM-induced osteoarthritis mouse model and osteochondral lesioned human explants
Source: Osteoarthr Cartil Open. 2026 Jun 5;8(3):100833. doi: 10.1016/j.ocarto.2026.100833 (PMC13276571; doi:10.1016/j.ocarto.2026.100833)
Supplement: Multimedia component 1 [file mmc1.docx]

**Supplementary Materials and Methods**

**Material and Methods**

***In vivo* study design**

For the *in vivo* experiment, 28 male 12 week-old C57BL/6J mice were purchased from Charles River Laboratories (Charles River, Chatillon-sur-Chalaronne, France). The animal procedures were all conducted at the Leiden University Medical Center and were approved by the Animal Welfare Committee (IvD) under number AVD1160020171405- PE.18.101.006 and in line with ARRIVE guidelines 2.0. Mice were housed in groups of four per polypropylene cage under a 12-hour light/dark cycle with ad libitum access to standard chow and water. The sample size was determined based on a priori power calculations and is consistent with previous DMM studies, providing a statistical power of 0.8 to detect biologically relevant differences in cartilage damage. The first group served as a sham (positive) control. The remaining 24 mice underwent surgery for destabilization of the medial meniscus (DMM) to establish a knee OA model as described elsewhere, Thirty min before surgery, pre-operative analgesia was administered by sub-cutaneous injection of Buprenorphine HCl (Vetergesic; Alstoe Animal Health, York, UK). The animal was then placed under isoflurane anaesthesia (4–5 % upon induction, 1–2 % for maintenance) and the incision for surgery was started on the right knee when the animal no longer displayed reflexes while the breathing was constant. A 1-cm longitudinal medial para-patellar incision was made to expose the knee joint. Subsequently, the knee joint was opened gently through lateral dislocation of the patella and patellar ligament and the medial meniscotibial ligament which anchors medial meniscus to the tibial plateau was cut. Successful destabilization of the medial meniscus was confirmed during surgery. After transection, the knee joint capsule was closed with a 6-0 absorbable suture and the skin-incision was closed with biological glue. Mice were immediately transferred to a warm post-operative recovery room. Within 48 hr post-surgery all animals received buprenorphine HCl sub-cutaneously every 8 hr. The mice were monitored daily to confirm their general health indicators according to their body weight and knee diameter Twenty-one days after surgery, the DMM mice were treated with a one-time intra-articular injection of 5 µL PBS (30 G needle), hydrogel, IOP either or not with hydrogel as illustrated in **Figure 1**. Animals were randomly allocated to the experimental groups using a dice. Moreover, to ensure blinded treatments, intra-articular (i.a.) injections were performed by a colleague not involved in randomization while the injection samples were prepared and coded by another colleague not involved in the animal experiment. At 35 days post-treatment, mice were euthanized by carbon dioxide (CO₂) inhalation in accordance with the EU Directive 2010/63/EU, Annex IV, on the protection of animals used for scientific purposes and as approved by the IvD (number AVD1160020171405- PE.18.101.006).

**Micro computed tomography measurements**

At 35 days after i.a. administration of the treatment groups, mice were euthanized by continuous CO2 inhalation, the right knee joints were harvested, and fixed with 4 % paraformaldehyde. After fixation for 48 h, the specimens were transferred to 70 % ethanol for high-resolution micro computed tomography (Micro-CT) (Skyscan 1072, Skyscan, Aartselaar, Belgium). The scanner was set at a gamma-ray voltage of 50 KV and a current of 200 uA, 0.5  mm Al filter, and a resolution of 9 μm per pixel. Subchondral bone morphology were analyzed using 3D data analysis software (CTAnalyzer, Skyscan). The region of interest (ROI) was defined within the subchondral bone of the medial tibial plateau and comprised 15 consecutive cross-sections, corresponding to a total ROI thickness of 135 µm. Quantitative parameters included bone volume fraction (BV/TV), trabecular thickness (Tb.Th), trabecular number (Tb.N), and trabecular separation (Tb.Sp) (1).

**(Immuno-) histochemistry**

Knee joints were fixed in 4 % paraformaldehyde (24 h), decalcified using a commercial mol-decalcifier (Milestone; pH 7.4) for 5 days at 37 °C, embedded in paraffin, and sectioned at 5 μm. Sections were stained with Hematoxylin & Eosin (H&E) and Safranin O/Fast green. Structural damage of cartilage was assessed using a structural cartilage damage scoring system, as outlined previously (2, 3). This modified damage scoring focuses on structural features of cartilage degeneration, including surface integrity, cartilage thickness, and chondrocyte organization in the medial femoral condyle and medial tibial plateau (3). Importantly, comparison with traditional OARSI scoring in meniscal cartilage demonstrated a strong positive correlation (Spearman’s r = 0.76, *P* ≤ 0.01), indicating that the damage scoring system provides a reliable and comparable alternative to standard OARSI assessment (3)

Osteophyte formation was graded histologically for each knee joint quadrant using a semi-quantitative scoring system based on the presence of chondro-osteophytes at the inner and/or outer joint margins (Grade 0 = no osteophytes; Grade 1 = osteophyte present at either inner or outer joint margin; Grade 2 = osteophytes present at both inner and outer joint margins), as previously described (4). Furthermore, total osteophyte area was quantified using ImageJ based image analysis (5). Because the most severe changes were consistently found on the tibial side of the joints, the analysis was focused on the tibial plateaus. Synovitis was evaluated on histological knee sections using a semi-quantitative scoring system adapted from standardized murine OA synovitis assessment guidelines, based on synovial lining layer hyperplasia and stromal cellularity in the medial tibial (MT), medial femoral (MF), lateral tibial (LT), and lateral femoral (LF) compartments (6).

Immunohistochemistry (IHC) staining was performed on knee joint sections for collagen type 2 (Col2), Mmp13 and CCDC80. For antibody staining endogenous peroxidase activity was blocked by MeOH/0.3 % H2O2. Subsequently, antigen retrieval was performed with Proteinase K (25 ug/ml) followed by hyaluronidase (5 mg/mL). Sections were blocked with 5 % PBS-BSA and incubated overnight at 4 °C with the following primary antibodies: Col2 mouse monoclonal antibody (2  μg/mL , ab34712, Abcam), Mmp13 monoclonal antibody (2  μg/mL, SC-515284, Santa Cruz Biotechnology), and CCDC80 polyclonal antibody (2  μg/mL, A65521, Thermo Fisher Scientific). The next day, the sections were incubated with Powervision-Poly/HRP (ImmunoLogic), followed by incubation with DAB (Sigma). Sections were dehydrated with ethanol (50-100 %) and Histoclear and mounted with Pertex. Quantification of image intensity was performed using ImageJ, as described elsewhere (7).

***Ex vivo* human lesioned explant experiment**

**Study design and culture components**

Osteochondral explants were collected from macroscopically preserved and lesioned areas from joints obtained from OA patients in the RAAK study (8), as outlined extensively previously (9) . The RAAK-study is aimed at biobanking of joint materials of patients who underwent a total joint replacement surgery due to OA. The RAAK-study is approved by the medical ethics committee of the Leiden University Medical Center (P19.013) and informed consent was obtained from subjects.A total of 38 osteochondral explants were obtained from six donors for this study and were divided into treatment groups as follows: control preserved (n=10 explants, N=6 donors), lesioned (n=11 explants, N=6 donors), and lesioned with IOP (n=16 explants, N=6 donors). Donor characteristics are provided in **Supplementary Table S2**. After harvesting, the explants were first washed with PBS and taken into culture in chondrogenic differentiation medium (CDM) in a 5 % CO2 incubator at 37 °C. The medium was refreshed every three days measurements.

**IOP Treatment**

IOP treatment for the lesioned explants began on day 3, with 100 µM of IOP (10) added to the medium on days 3, 6, and 9, as illustrated in **Figure 1-B**. On day 12, cartilage and bone were separated using a scalpel, snap frozen in liquid nitrogen and stored at 80 °C for RNA isolation. For histology, a part was fixed in 4% formaldehyde. Medium was collected on day 12 and stored at 80 °C.

**Determining cartilage integrity**

**Sulphated glycosaminoglycan (sGAGs) measurement**

Sulphated glycosaminoglycans (sGAGs) concentration was measured in conditioned media of explants collected from day 3, 6, 9 and 12 following extraction with the photometric 1,9 dimethylene blue (DMMB; Sigma-Aldrich) dye method (11). Shark chondroitin sulfate (Sigma-Aldrich) was used as the reference standard. To measure concentrations, 100 µl of medium or digested cartilage was mixed with 200 µl of DMMB solution and the absorbance at 525 nm and 595 nm was measured in a microplate reader (Synergy HT; BioTek, Winooski, USA).

**(Immuno-) histochemistry of Osteochondral explants**

Osteochondral explants were fixed in 4 % formaldehyde for 48 h and decalcified using mol-decalcifier (Milestone) for one weeks at 37 °C. Subsequently, samples were dehydrated with an automated tissue processing apparatus and embedded in paraffin. Tissue sections of 5 μm were stained with Hematoxylin and Eosin (H&E), toluidine blue (Sigma-Aldrich), safranin-O/Fast Green, and mounted with Pertex (Sigma-Aldrich). Quantification of OA related cartilage damage was scored according to Mankin et al . Immunohistochemical (IHC) staining for collagen type II (Col2) was performed on joint sections using the same antibody concentration and protocol as described for the *in vivo* experiments above. Col2 staining was evaluated independently by two blinded observers. Staining intensity was scored separately for cytoplasmic and nuclear localization of chondrocytes within each cartilage layer (superficial, middle, and deep). Scores of 0, 1, and 2 were assigned for absent, moderate, and strong staining intensity, respectively. The scores from all layers were summed, resulting in a total score ranging from 0 (no staining in any cartilage layer) to 12 (strong staining in both cytoplasm and nuclei across all cartilage layers). RNA isolation, Reverse Transcription and quantitative Real-Time PCR. Cartilage compartments were manually separated and were lysed using Trizol (Invitrogen) and stored at −80 °C until further processing. RNA was isolated from the samples using the RNeasy Mini Kit (Qiagen). cDNA synthesis was performed using the First Strand cDNA Synthesis Kit (Roche Applied Science). Subsequently, RT-qPCR was performed using SYBR Green without the ROX reference dye (Roche Applied Science) and the QuantStudio 6 Real-Time PCR system (Applied Biosystems). Primer sequences used are listed in Supplementary Table 3. GAPDH and SDHA were used as housekeeping genes. The measured gene expression levels were corrected for the housekeeping genes GAPDH and SDHA, and the foldchanges were calculated using the 2–∆∆CT method.

**Supplementary Figures**


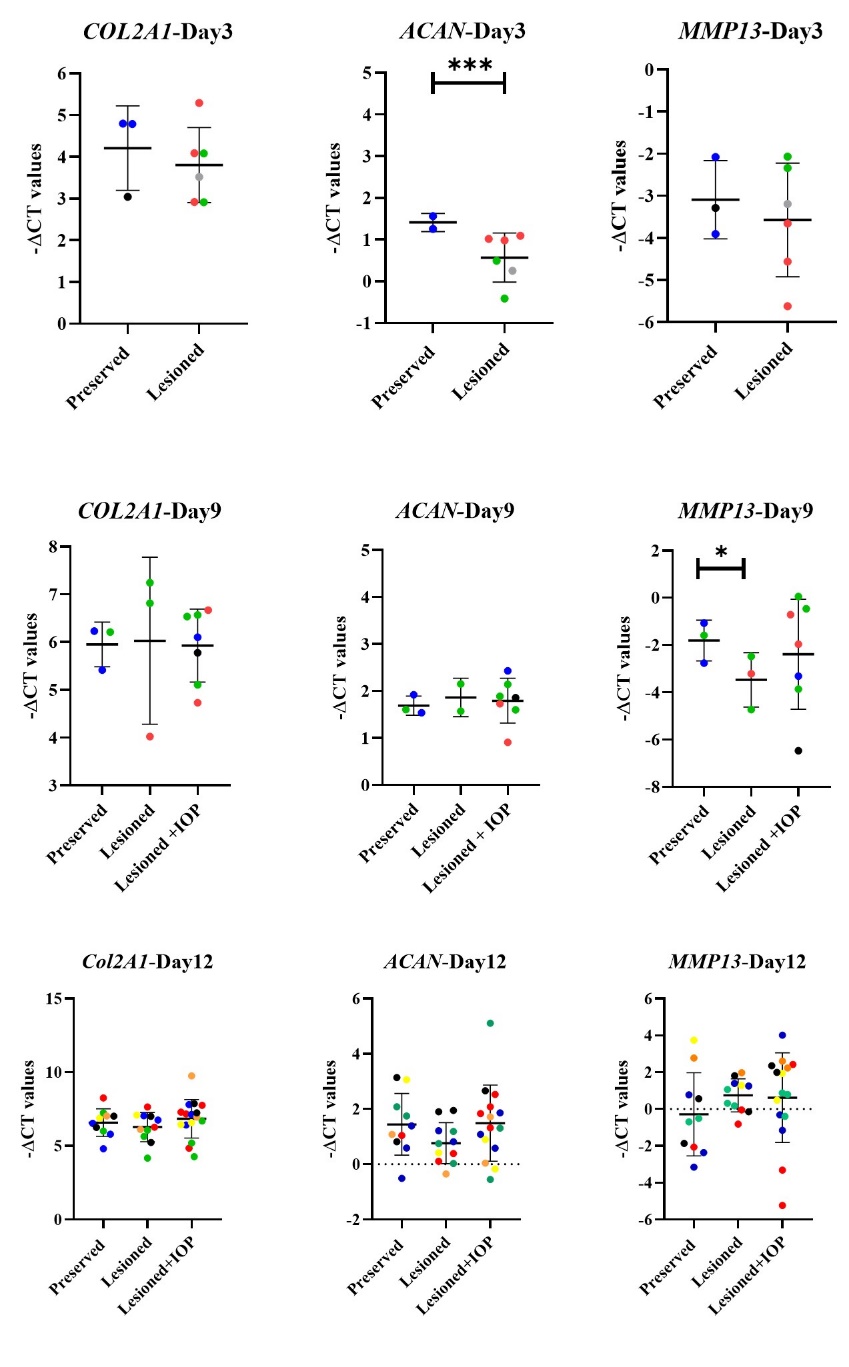


**Supplementary Figure S1. Gene expression analysis by RT-qPCR at day 3, 9 and 12 for *COL2A1*, *ACAN*, and *MMP13***, presented as −ΔCT values for preserved, lesioned, and IOP-treated lesioned explants. Statistically significant differences are indicated by * where *P* ≤ 0.05, ***P* ≤ 0.01 between the indicated groups.

**Supplementary Tables:**

**Supplementary Table S1**. ***In vivo* experimental groups**

| Description | DMM | Hydrogel | IOP | Number |
| --- | --- | --- | --- | --- |
| Sham control | - | - | - | 6 |
| DMM+PBS (Control OA) | + | - | - | 6 |
| DMM+Hydrogel | + | + | - | 6 |
| DMM+IOP | + | - | + | 6 |
| DMM+IOP-Hydrogel | + | + | + | 6 |

DMM=Destabilization of medial meniscus, IOP= Iopanoic acid

**Supplementary Table S2**. **Baseline information of the donors included in the *ex vivo* experiment this study.**

| Characteristic |  | Average±SD [Range] |
| --- | --- | --- |
| Age |  | 75.83±10.57 [57-87] |
| Sex |  | 3M, 3F |
| BMI |  | 30.83±3.067 [28-36] |

The table represents the age, sex, and BMI of donors used in this study. Legend: F=Female; age given in years)

**Supplementary Table S3. Primer sequences used in RT-qPCR**

| **Primers** | **Forward primer (5'- 3')** | **Reverse primer (5'- 3')** |
| --- | --- | --- |
| *GADPH* | 5'-TGCCATGTAGACCCCTTGAAG-3' | 5'-ATGGTACATGACAAGGTGCGG-3' |
| *SDHA* | 5'-TGGGAACAAGAGGGCATCTG-3' | 5'-GCCTACCACCACTGCATCAA-3' |
| *COL2A1* | 5'-CTACCCCAATCCAGCAAACGT-3' | 5'-AGGTGATGTTCTGGGAGCCTT-3' |
| *MMP13* | 5'-TTGAGCTGGACTCATTGTCG-3' | 5'- GGAGCCTCTCAGTCATGGAG-3' |
| *ACAN* | 5'-AGAGACTCACACAGTCGAAACAGC-3' | 5'-CTATGTTACAGTGCTCGCCAGTG-3' |

**References:**

1. Botter S, Glasson S, Hopkins B, Clockaerts S, Weinans H, Van Leeuwen J, et al. ADAMTS5−/− mice have less subchondral bone changes after induction of osteoarthritis through surgical instability: implications for a link between cartilage and subchondral bone changes. Osteoarthritis and cartilage. 2009;17(5):636–45.

2. Armstrong AR, Carlson CS, Rendahl AK, Loeser RF. Optimization of histologic grading schemes in spontaneous and surgically-induced murine models of osteoarthritis. Osteoarthritis and cartilage. 2021;29(4):536–46.

3. Sayedipour SS, Nikkels J, Tertel T, Suchiman HE, Koedam M, Balbi M, et al. Therapeutic efficacy of extracellular vesicles from hiPSC-derived MSCs in serum-containing and xeno-free media for osteoarthritis treatment. Stem Cell Research & Therapy. 2026;17(1):72.

4. Grote CW, Mackay MJ, Liu X, Lu Q, Wang J. A modified comprehensive grading system for murine knee osteoarthritis: scoring the whole joint as an organ. Osteoarthritis and Cartilage. 2022;30:S95.

5. Ozkan H, Di Francesco M, Willcockson H, Valdés-Fernández J, Di Francesco V, Granero-Moltó F, et al. Sustained inhibition of CC-chemokine receptor-2 via intraarticular deposition of polymeric microplates in post-traumatic osteoarthritis. Drug Delivery and Translational Research. 2023;13(2):689–701.

6. Obeidat AM, Kim SY, Burt KG, Hu B, Li J, Ishihara S, et al. A standardized approach to evaluation and reporting of synovial histopathology in two surgically induced murine models of osteoarthritis. Osteoarthritis and cartilage. 2024;32(10):1273–82.

7. Bloks NG, Harissa Z, Mazzini G, Adkar SS, Dicks AR, Hajmousa G, et al. A Damaging COL6A3 Variant Alters the MIR31HG‐Regulated Response of Chondrocytes in Neocartilage Organoids to Hyperphysiologic Mechanical Loading. Advanced Science. 2024;11(36):2400720.

8. Ramos YF, den Hollander W, Bovee JV, Bomer N, van der Breggen R, Lakenberg N, et al. Genes involved in the osteoarthritis process identified through genome wide expression analysis in articular cartilage; the RAAK study. PloS one. 2014;9(7):e103056.

9. Houtman E, Tuerlings M, Riechelman J, Suchiman EH, van der Wal RJ, Nelissen RG, et al. Elucidating mechano-pathology of osteoarthritis: transcriptome-wide differences in mechanically stressed aged human cartilage explants. Arthritis Research & Therapy. 2021;23(1):215.

10. Houtman E, Tuerlings M, Suchiman HED, Lakenberg N, Cornelis FM, Mei H, et al. Inhibiting thyroid activation in aged human explants prevents mechanical induced detrimental signalling by mitigating metabolic processes. Rheumatology. 2023;62(1):457–66.

11. RW F. Improved quantitation and discrimination of sulphated glycosaminoglycans by use of dimethylmethylene blue. Biochim Biophys Acta. 1986;883:173–7.
